# Division of Labor by the HELQ, BLM, and FANCM Helicases during Homologous Recombination Repair in *Drosophila melanogaster*

**DOI:** 10.3390/genes13030474

**Published:** 2022-03-08

**Authors:** Adam Thomas, Julie Cox, Kelly B. Wolfe, Carrie Hui Mingalone, Haleigh R. Yaspan, Mitch McVey

**Affiliations:** Department of Biology, Tufts University, Medford, MA 02155, USA; amthomas1024@gmail.com (A.T.); jul.cox007@gmail.com (J.C.); kelly.m.beagan@gmail.com (K.B.W.); carrie.huimingalone@gmail.com (C.H.M.); haleighrwilliams@gmail.com (H.R.Y.)

**Keywords:** double-strand break, D-loop, mutagenesis, end joining, transposon

## Abstract

Repair of DNA double-strand breaks by homologous recombination (HR) requires a carefully orchestrated sequence of events involving many proteins. One type of HR, synthesis-dependent strand annealing (SDSA), proceeds via the formation of a displacement loop (D-loop) when RAD51-coated single-stranded DNA invades a homologous template. The 3′ end of the single-stranded DNA is extended by DNA synthesis. In SDSA, the D-loop is then disassembled prior to strand annealing. While many helicases can unwind D-loops in vitro, how their action is choreographed in vivo remains to be determined. To clarify the roles of various DNA helicases during SDSA, we used a double-strand gap repair assay to study the outcomes of homologous recombination repair in *Drosophila melanogaster* lacking the BLM, HELQ, and FANCM helicases. We found that the absence of any of these three helicases impairs gap repair. In addition, flies lacking both BLM and HELQ or HELQ and FANCM had more severe SDSA defects than the corresponding single mutants. In the absence of BLM, a large percentage of repair events were accompanied by flanking deletions. Strikingly, these deletions were mostly abolished in the *blm helq* and *blm fancm* double mutants. Our results suggest that the BLM, HELQ, and FANCM helicases play distinct roles during SDSA, with HELQ and FANCM acting early to promote the formation of recombination intermediates that are then processed by BLM to prevent repair by deletion-prone mechanisms.

## 1. Introduction

Homologous recombination (HR) is a high-fidelity mechanism of DNA double-strand break repair. In eukaryotes, the initial steps of HR involve 3′ to 5′ resection of DNA ends by an orchestrated collection of nucleases and helicases. The RAD51 protein is loaded onto the resultant single-stranded DNA (ssDNA) and the nucleoprotein filament promotes a search for homologous repair templates, such as a sister chromatid, homologous chromosome, or ectopic homologous sequence. RAD51-coated ssDNA invades the template and forms a displacement loop (D-loop) intermediate, which is then extended by DNA polymerase delta [1], with possible contributions from translesion polymerases eta and zeta [2,3,4,5,6,7]. The D-loop enlarges until it is about 100–200 nt in size, at which time it converts to a migrating D-loop as synthesis proceeds [6]. In the synthesis-dependent strand annealing (SDSA) model of HR, the D-loop is unwound by one or more DNA helicases, and the nascent DNA anneals to complementary sequences on the other side of the break [8]. Repair concludes with fill-in synthesis and ligation. Importantly, SDSA proceeds without the formation of Holliday junctions, thereby preventing mitotic crossovers that can lead to the loss of heterozygosity (reviewed in [9,10,11]).

While the proteins that carry out the initial steps of HR, such as DNA resection and strand invasion, have been extensively characterized, the factors involved in processes that occur downstream of initial D-loop formation are not as well understood, particularly in metazoans. This is especially true for DNA helicases that promote D-loop enlargement, migration, and disassembly. Much of our current understanding comes from in vitro assays and from elegant studies in budding and fission yeasts, *Saccharomyces cerevisiae* and *Schizosaccharomyces pombe*. Several yeast DNA helicases can unwind D-loops in vitro, including Mph1/Fml1 [2,12,13,14], Srs2 [15], and the Sgs1-Top3-Rmi1 helicase-topoisomerase complex [16]. These proteins also promote SDSA in vivo [14,17], with Srs2 and Mph1/Sgs1-Top3-Rmi1 acting in two distinct pathways to promote D-loop disruption [18].

Biochemical and genetic experiments have identified several functional mammalian homologs of these yeast helicases that can unwind D-loops, including FANCM, BLM, and RTEL [19,20,21,22]. In addition, two helicases with roles in SDSA and D-loop unwinding have been identified in *Drosophila melanogaster*: BLM [23,24,25,26], and FANCM [27,28]. Loss of Drosophila BLM results in a severe defect in SDSA that is often accompanied by deletions flanking the double-strand break [25], while loss of FANCM results in a mild SDSA defect, consistent with the limited ability of the purified protein to unwind D-loop structures [28].

In addition to the helicases mentioned above, the HelQ helicase appears to be important for homologous recombination repair of double-strand breaks. HelQ promotes homologous recombination downstream of strand invasion in both *Caenorhabditis elegans* [29,30] and mammals [31,32,33], where its mechanism of action appears to be via an interaction with RAD51 paralogs. Drosophila HELQ is also important for double-strand break repair by homologous recombination [34]. Flies lacking HELQ, similar to flies with mutations in genes encoding RAD51 paralogs, have impaired meiotic recombination and eggshell defects that can be reversed by preventing meiotic double-strand breaks [35].

Here, we have used a double-strand gap repair assay to study the outcomes of HR repair in the male germline of *Drosophila* lacking the BLM, HELQ, and FANCM helicases singly or in combination. We find that the absence of any of these three helicases impairs HR, as has been previously shown. Flies lacking both BLM and HELQ or HELQ and FANCM have more severe gap repair defects than the corresponding single mutants, suggesting that these three helicases act independently and/or at different stages of HR. In the absence of BLM, a large percentage of repair events are accompanied by flanking deletions. Strikingly, these deletions are suppressed in the *blm helq* and *blm fancm* double mutants. Thus, HELQ and FANCM may promote the formation of a repair intermediate that must be disassembled by the BLM to prevent processing by deletion-forming mechanisms.

## 2. Materials and Methods

**Generation of mutants:** The *helq*^288*A*^ mutant was created by imprecise excision of *P*{*SUPor-P*}*mus301^KG^*^09098^, a *P* element inserted in the fourth intron of *spn-*C/*mus301*. The excision resulted in a 2069 bp upstream deletion, removing the second, third, and fourth exons, as well as part of the first. Of the *P* element sequence, 494 bp remain inserted at the deletion site. Although the promoter, transcription, and translation start sites are unaffected, all conserved helicase motifs are deleted.

The *blm^N^*^1^ and *blm^N^*^2^ mutants were created by imprecise excision of *P*{*EPgy2*}*Blm^EY^*^03745^ [23]. The *blm^D^*^2^ allele is described in [36]. The *spn-A* mutants used in the mutagen sensitivity assays are null mutations. *spn-A*^093^ is a nonsense mutation in the N-terminal portion of the protein, while *spn-A*^057^ is a missense mutation in the central region of the protein [37].

The *fancm*^0693^ null mutant is described in [27]. To prevent inaccurate results due to second site mutations, the *fancm*^0693^ allele was always placed in trans to *Df(3R)ED6058,* which deletes approximately 423 kilobases of chromosome *3*, including the *FANCM* locus. A dominant *Drop* allele (*Dr*) was crossed onto the *Df(3R)ED6058* chromosome by meiotic recombination to allow for positive identification of the deficiency chromosome during mating schemes.

**Mutagen sensitivity assays:** Sensitivity assays tested the ability of larvae to survive exposure to mutagenic chemicals and eclose as adults. Five to eight female flies heterozygous for the mutation of interest were crossed to three heterozygous mutant males. The females laid eggs for three days, and the parents were moved into new vials for two additional days. The first set of vials was treated with mechlorethamine (nitrogen mustard, Sigma) or topotecan (Enzo Life Sciences), while the second set was treated with a vehicle (water). Between two and six replicates (each with five to ten individual vials) for each dose of mutagen were conducted. Relative survival was calculated by comparing the percentage of homozygous adult offspring in treated vials to the percentage of homozygous adult offspring in untreated vials.

***P{w^a^}* assay:** The *P{w^a^}* construct contains a functional copy of the *white* gene disrupted by the *copia* retrotransposon [38]. Flies homozygous for the construct have apricot-colored eyes because of the *copia* insertion, which impairs the splicing and transcription of the *white* gene. The entire *P{w^a^}* construct is inserted into an intron of *scalloped*, an essential gene located on the *X* chromosome. Excision of *P{w^a^}* leaves a 14 kb gap with non-complementary 17 nucleotide 3′ overhangs. Repair events fall into three categories: complete (entire *P{w^a^}* construct restored), SDSA + LTR annealing (complete synthesis of *white*, annealing at *copia* LTRs), and aborted SDSA (failure to initiate synthesis or synthesis aborted before reaching the *copia* LTRs).

Mutant male flies carried (1) a single copy of the *P{w^a^}* construct on the *X* chromosome, (2) a transposase (*H*{*w*^+^, *Δ2-3*}*Hop2.1*) on chromosome *2*, and (3) *mus301*, *blm, fancm,* or pairwise combinations of these mutationson chromosome *3*. They were mated to females that were homozygous for *P{w^a^}* but were otherwise wild type. Repair events from the pre-meiotic male germline were recovered in the following generation. All F_1_ female offspring inherited one wild-type maternal copy of *P{w^a^}* and one paternal copy of *P{w^a^}*. If the paternal copy was not excised or was repaired completely, the female offspring had two complete copies of *P{w^a^}* and apricot eyes. Most female offspring fell into this category, likely because the transposon was not excised from the paternal *X* chromosome. If the paternal copy was repaired with full synthesis of *white* and annealing at the LTRs, the female offspring had red eyes. If repair from either end of the break did not initiate, or did not reach, the LTRs, the female offspring had yellow eyes resulting from the single intact maternal copy of *P{w^a^}*. In this way, the relative frequency of SDSA and aborted SDSA in the different mutant backgrounds was determined. All mutant backgrounds were compared to a wild-type control, where males carried only the *P{w^a^}* transposon and *H*{*w*^+^, *Δ2-3*}*Hop2.1* transposase.

In addition to the relative frequencies of repair pathways, the extent of synthesis in the aborted SDSA repair category was also determined. White-eyed F_2_ male progeny of the F_1_ yellow-eyed females that inherited the aberrantly repaired *P{w^a^}* construct were recovered, and subsequent PCR analysis was used to determine the extent of repair synthesis for each event.

Repair events occasionally resulted in flanking deletions, which included both lethal and non-lethal varieties. Deletions that extended into the coding region of the *scalloped* resulted in F_1_ female progeny with misshapen wings and/or an absence of F_2_ white-eyed male progeny. Either of these phenotypes indicates a lethal flanking deletion. Additionally, non-lethal deletions could remove some *scalloped* intron sequences but not extend into an exon. Analysis of small deletions was performed using a series of primers that anneal to the *scalloped* intronic sequence. Statistical analysis of repair event frequencies, extension of repair synthesis, and deletion frequencies was performed in GraphPad Prism 9.

## 3. Results

### 3.1. HELQ Is Required for Homologous Recombination Repair of Double-Strand Breaks

**Figure 1:**
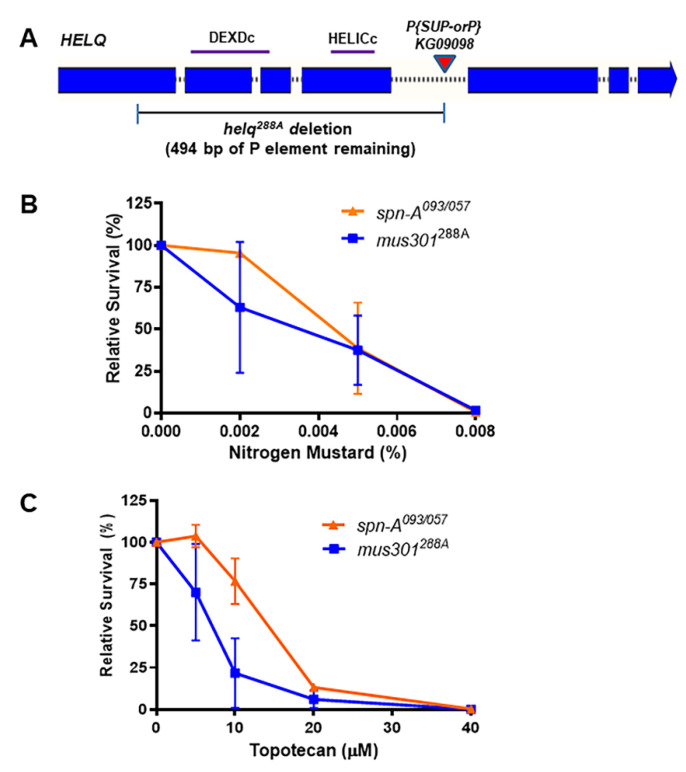
Drosophila *helq* mutants are sensitive to nitrogen mustard and topotecan. (**A**) *helq*^288*A*^ allele created through imprecise *P* element excision. (**B**) Nitrogen mustard sensitivity of the *helq* and *spn-A* mutants. (**C**) Topotecan sensitivity of the *helq* and *spn-A* mutants. Relative survival is calculated as the number of homozygous mutants surviving in treated vs. control vials compared to the total number of flies in each vial. Each data point consists of 2–3 trials of 5–8 vials. Means and standard deviations are shown.

*Drosophila* HELQ (encoded by the *spn-C*/*mus301* gene) is required for survival following exposure to methyl methanesulfonate and nitrogen mustard and for the repair of meiotic double-strand breaks [34,39]. To determine whether HELQ is required for double-strand break repair in mitotic cells, we created a deletion mutation of *HELQ* by imprecise excision of *P(SUPor-P)mus301^KG^*^09098^. The *helq*^288*A*^ mutation removes more than 2 kilobases of coding sequence, including the entire helicase domain (Figure 1A). We confirmed that *helq* mutants are sensitive to nitrogen mustard at a level comparable to homologous recombination-deficient *spn-A* mutants lacking the RAD51 recombinase (Figure 1B). We then tested the *helq* mutant for sensitivity to topotecan, a topoisomerase I inhibitor that induces one-ended DNA double-strand breaks in replicating cells. Flies lacking HELQ were hypersensitive to topotecan, with relative survival slightly less than *spn-A* mutants (Figure 1C).

**Figure 2:**
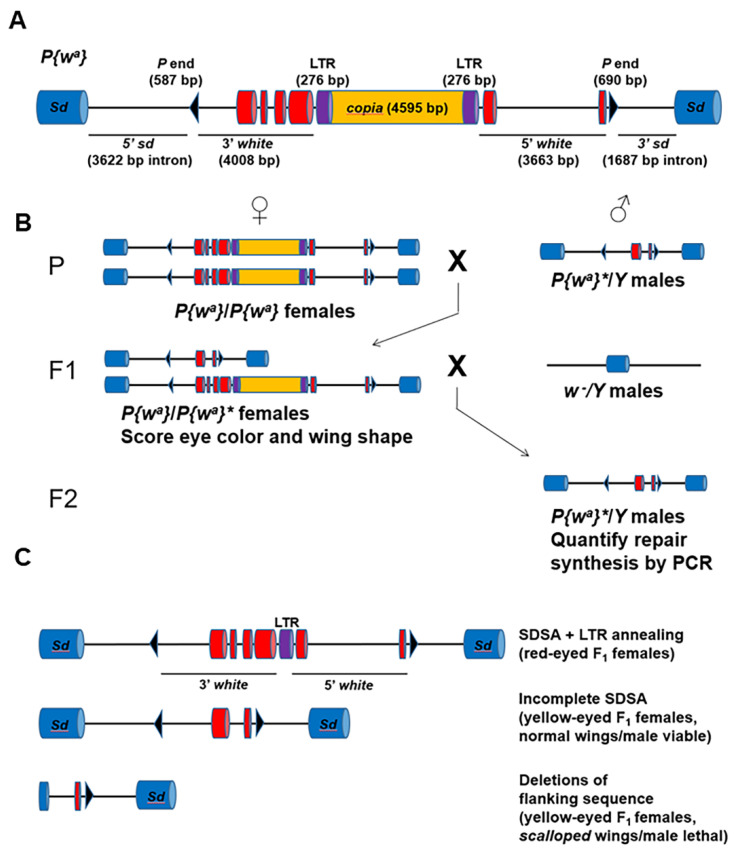
The *P{w^a^}* gap repair assay. (**A**) The *P{w^a^}* transposon, located on the *X* chromosome, contains a complete copy of the *white* gene interrupted by the *copia* retrotransposon. The presence of *copia* reduces the expression of *white*, resulting in yellow-eyed females if present in one copy and apricot-eyed females if present in two copies. (**B**) *P*-element excision is stimulated in males in the presence of *P* transposase, creating a 14 kilobase double-stranded gap. Repair events occurring in the male pre-meiotic germline (P{w^a^}*) are recovered in F_1_ females with an intact copy of *P{w^a^}*. The amount of repair synthesis that occurred during SDSA is quantified by PCR using genomic DNA from white-eyed F_2_ male progeny. (**C**) HR repair involving two-ended strand invasion into an uncut sister chromatid, synthesis, and annealing at the *copia* LTRs deletes most of *copia*, restoring *white* expression and resulting in red-eyed female progeny (top). Aborted HR repair in which end-joining occurs prior to synthesis of *white* results in yellow-eyed female progeny (middle). Repair involving deletions into flanking *sd* sequences results in scalloped-winged females and/or lethality in F_2_ males (bottom).

To further investigate the role of HELQ in HR repair, we tested *helq* mutants using a well-characterized gap repair assay [40]. In this assay, a 14 kilobase *P* element carrying the *white* gene is inserted on the *X* chromosome (Figure 2A). The 5′ and 3′ regions of *white* are interrupted by a *copia* retrotransposon, which decreases *white* expression so that female progeny with two copies of *P{w^a^}* have apricot-colored eyes and females with one copy have yellow eyes. Expression of transposase causes infrequent excision of the *P* element, creating a 14 kilobase double-stranded gap relative to the uncut sister chromatid. Excision of *P{w^a^}* and repair can occur in both somatic and germline tissues.

Repair pathway choice is easily quantified by mating males undergoing excision and repair in their germlines to females possessing two copies of the *P{w^a^}* element, then scoring eye color in the F_1_ female progeny (Figure 2B). Strand invasion can occur on both sides of the gap, resulting in two D-loops that migrate toward each other as repair synthesis proceeds. Bidirectional synthesis past the 276 base-pair long terminal repeats (LTRs) of *copia* and annealing at the LTRs removes most of *copia*, creating a product with increased *white* expression (SDSA + LTR annealing; Figure 2C). For unknown reasons, synthesis sometimes aborts, and repair concludes through a DNA ligase 4-independent alternative end-joining process [41] (aborted SDSA; Figure 2C). Red-eyed F_1_ female progeny inherit SDSA + LTR annealing products that involve at least 4 kilobases of synthesis on each side, while yellow-eyed female progeny inherit products that result from aborted SDSA and alternative end joining.

**Figure 3:**
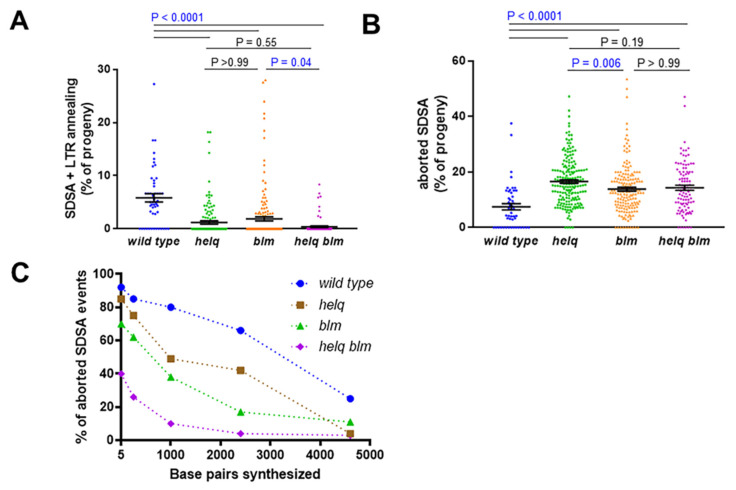
Both HELQ and BLM promote SDSA and long-distance repair synthesis. (**A**) Percentage of SDSA + LTR annealing in repair events observed in F_1_ females. (**B**) Percentage of aborted SDSA repair events observed in F_1_ females. For A and B, mean frequencies and standard errors are shown; each point represents progeny derived from a single male germline. Significance was calculated using a Kruskal–Wallis test with Dunn’s multiple comparisons post-test. Wild type (n = 50), *helq*^288*A*^ (n = 188), *blm^N^*^1^ (n = 170), *helq*^288*A*^ *blm^N^*^1^ (n = 101). (**C**) Minimum amount of DNA synthesized during aborted SDSA repair, measured from the right end of the break created following *P{w^a^}* excision. Wild type (n = 74), *helq*^288*A*^ (n = 67), *blm^N^*^1^ (n = 71), *helq*^288*A*^ *blm^N^*^1^ (n = 70). Statistical comparisons between all mutants can be found in Supplemental Tables S1–S3.

Males lacking HELQ showed a significant decrease in SDSA + LTR annealing repair events compared to the wild type, with a corresponding increase in end-joining repair following aborted HR (Figure 3A,B). This, combined with the topotecan sensitivity of *helq* mutants, suggests that HELQ plays an important mitotic role in the HR repair of double-strand breaks. To determine the extent of repair synthesis that occurred during incomplete SDSA in wild type and *helq* mutants, we performed PCR on genomic DNA isolated from F_2_ white-eyed males. Interestingly, *helq* mutant males had decreased repair synthesis at all distances measured (Figure 3C), suggesting that HELQ is important for processive synthesis during gap repair.

### 3.2. HELQ Plays a Role in SDSA Distinct from That of the BLM Helicase

The decreased use of SDSA + LTR annealing repair and shorter repair synthesis tracts observed for *helq* mutants mimic the phenotypes previously observed in flies lacking BLM [23,24]. Thus, to determine if BLM and HELQ perform different functions during HR, we tested *blm* single mutants and *helq blm* double mutants using the *P{w^a^}* assay. As previously reported, the *blm* mutants had a decreased percentage of SDSA + LTR annealing events and increased aborted SDSA repair compared to wild-type males (Figure 3A,B). Aborted SDSA repair events recovered from *blm* males also had shorter repair synthesis tracts than the wild type (Figure 3C). In the *helq blm* double mutants, we observed a significant decrease in SDSA + LTR annealing repair compared to the *blm* but not the *helq* mutants (Figure 3A). The levels of aborted SDSA repair were similar between both single mutants and the *helq blm* double mutant (Figure 3B). However, the repair synthesis tracts of the double mutants were significantly shorter than those of the single mutants at all but the longest distance (Figure 3C), indicating that HELQ and BLM either act at different stages of SDSA or are individually limiting for repair synthesis.

In *blm* mutants, aborted SDSA repair events are frequently accompanied by flanking deletions that impact the expression of the essential *sd* gene, resulting in a scalloped-wing phenotype and/or male lethality in the F_2_ generation [25] (Figure 2C). To analyze deletion frequency in various genetic backgrounds, we quantified the frequency of F_1_ scalloped-winged females and F_2_ male lethal deletions. In contrast to the *blm* mutants, we observed very few lethal deletions in the *helq* mutants (Table 1). Interestingly, there was partial suppression of both *scalloped* females and male-lethal deletions in the double mutant.

Smaller flanking deletions that extend into the *scalloped* intronic sequence but do not affect *scalloped* expression may be non-lethal and remain undetected by our deletion analysis in Table 1. A negative PCR result for the DNA synthesis analysis at 5 bp on the right end of *P{w^a^}* could indicate either a lack of synthesis from that end or a non-lethal flanking deletion. To discern between these two possibilities, we analyzed these events using primers that anneal to the *scalloped* intronic sequence, either to the left or the right of the insertion site. Similar to the large deletion frequency, small deletions were common in *blm* mutants but not in *helq* mutants (Table 2). In the *helq blm* double mutant, we again saw suppression of the *blm* phenotype. Together with the large deletion analysis, these data suggest that HELQ may promote the formation of an SDSA intermediate that requires BLM for its resolution. In the absence of BLM, deletion-prone repair predominates.

### 3.3. FANCM Helicase Has a Minor Role in SDSA

**Figure 4:**
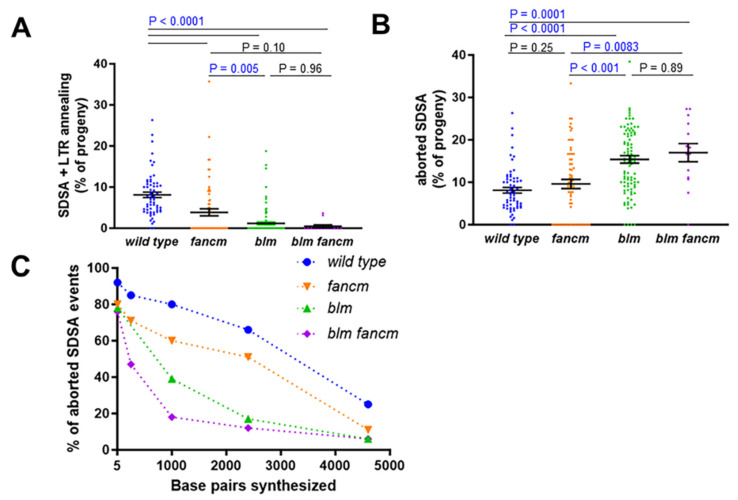
FANCM performs a minor role in SDSA compared to BLM. (**A**) Percentage of SDSA + LTR annealing in repair events observed in F_1_ females. (**B**) Percentage of aborted SDSA repair events observed in F_1_ females. For A and B, mean frequencies and standard errors are shown; each point represents progeny derived from a single male germline. Significance was calculated using a Kruskal–Wallis test with Dunn’s multiple comparisons. Wild type (n = 63), *fancm*^0693^/*Df(3R)ED6058* (n = 63), *blm^D^*^2^/*blm^N^*^2^ (n = 94), *fancm*^0693^ *blm^N^*^1^/*Df(3R)ED6058 blm^N^*^1^ (n = 14) (**C**) Minimum amount of DNA synthesized during aborted SDSA repair, measured from the right end of the break created following *P{w^a^}* excision. Wild type (Figure 3; n = 74), *fancm*^0693^/*Df(3R)ED6058* (n = 35), *blm^D^*^2^/*blm^N^*^2^ (n = 36), *fancm*^0693^ *blm^N^*^1^/*Df(3R)ED6058 blm^N^*^1^ (n = 17). Statistical comparisons between all mutants can be found in Supplemental Tables S1–S3.

The FANCM protein has also been shown to promote SDSA repair, although *fancm* mutant defects in the *P{w^a^}* assay are less severe than those in *blm* mutants [27]. To determine if the roles of FANCM and BLM in SDSA overlap, we compared *fancm* and *blm* single and double mutants using the *P{w^a^}* gap repair assay. The *blm* mutations used were *blm^N^*^1^*,* *blm^N^*^2^, and *blm^D^*^2^, all of which cause similar defects in SDSA repair [23]. As previously reported, *fancm* mutants had fewer SDSA + LTR repair events compared to the wild type, although the decrease was not as large as with the *blm* mutants [27] (Figure 4A). *blm* mutants also had a greater percentage of aborted SDSA repair products compared to *fancm* mutants (Figure 4B), and the amount of DNA synthesis from the right end of the gap was less in *blm* mutants than in *fancm* mutants (Figure 4C).

Interestingly, the percentages of SDSA + LTR annealing products and aborted SDSA products were similar in the *blm* and *blm fancm* mutants (Figure 4A,B). In addition, the amount of repair synthesis at all distances measured in the double mutant was not statistically different from that in the *blm* mutant (Figure 4C and Supplemental Table S3). To further probe the differences between the *fancm* and *blm* mutants, we quantified the percentage of deletion events in each of the single mutants and compared them to the *blm fancm* double mutant. In contrast to the *blm* mutant phenotype, the loss of *FANCM* did not significantly increase the frequency of repair events with large deletions (Table 3). Intriguingly, mutation of *FANCM* partially suppressed the deletion-prone repair of the *blm* mutants, similar to what we observed with the *helq blm* double mutants.

### 3.4. Loss of Both HELQ and FANCM Has Additive Effects on Repair Synthesis during SDSA

The data from the gap repair assay suggest that both HELQ and FANCM play roles distinct from the BLM helicase in SDSA repair. To determine whether HELQ and FANCM may also act independently from each other during HR, we constructed helq fancm double mutants and tested them in the P{wa} assay.

**Figure 5:**
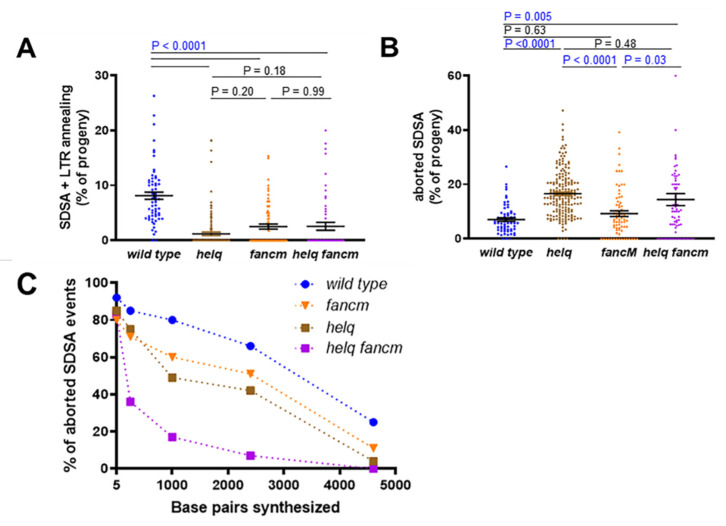
Additive effects of loss of HELQ and FANCM on SDSA repair synthesis. (**A**) Percentage of SDSA + LTR annealing in repair events observed in F1 females. (**B**) Percentage of aborted SDSA repair events observed in F1 females. For A and B, mean frequencies and standard errors are shown; each point represents progeny derived from a single male germline. Significance was calculated using a Kruskal–Wallis test with Dunn’s multiple comparisons. Wild type (Figure 4; n = 63), *helq*^288*A*^ (Figure 3; n = 188), *fancm*^0693^/*Df(3R)ED6058* (n = 91), *helq*^288*A*^ *fancm*^0693^/*helq*^288*A*^ *Df(3R)ED6058* (n = 71) (**C**) Minimum amount of DNA synthesized during aborted SDSA repair, measured from the right end of the break created following P{wa} excision. Wild type (Figure 3; n = 74), *helq*^288*A*^ (Figure 3; n = 67), *fancm*^0693^/*Df(3R)ED6058* (Figure 4; n = 35), *helq*^288*A*^ *fancm*^0693^*/helq*^288*A*^ *Df(3R)ED6058* (n = 51). Statistical comparisons between all mutants can be found in Supplemental Tables S1–S3.

While loss of either helicase resulted in a significant decrease in red-eyed progeny compared to the wild type, mutation of *HELQ* caused a more severe SDSA defect compared to mutation of *FANCM* (Figure 5A). There was no additional significant decrease in red-eyed progeny in the *helq fancm* double mutant compared to the *helq* mutant, nor was the frequency of aborted SDSA further elevated in the *helq fancm* double mutant compared to the *helq* mutant (Figure 5B). However, when repair synthesis in the aborted SDSA events was quantified by PCR, we observed significantly shorter repair synthesis tracts in the *helq fancm* double mutant compared to either single mutant (Figure 5C and Supplemental Table S3). This suggests that HELQ and FANCM may have non-overlapping roles during SDSA. Alternatively, each protein may be limiting for a process that promotes repair synthesis.

## 4. Discussion

In this study, we characterized defects in the SDSA repair pathway in flies lacking three different DNA helicases. We confirmed reports that BLM and FANCM helicases are important for SDSA, with *blm* mutants affecting repair synthesis during SDSA to a greater extent than *fancm* mutants [23,27]. Previously, the Sekelsky lab suggested that the milder phenotypes of *fancm* mutants could be explained by two scenarios. First, FANCM might assist in the recruitment of the BLM-TOP3a complex to D-loop structures, where it could promote dissociation. Second, FANCM might act to unwind small D-loops that form when SDSA is initiated, but not larger D-loops that are created as SDSA proceeds [27,28]. Our examination of gap repair in *blm fancm* double mutants showed mostly non-additive effects of the *blm* and *fancm* mutations, consistent with the proteins having at least partially overlapping functions. However, these genetic data are not sufficient to distinguish between the two models presented above.

We also found that Drosophila HELQ helicase plays an important role in SDSA repair of mitotic double-strand breaks. HELQ was originally identified in a genetic screen for maternal effect mutants having altered eggshell morphology and was named *spn-C* [42]. Follow-up work showed that HELQ is needed for the repair of meiotic double-strand breaks and for resistance to the alkylating agent methyl methanesulfonate [34]. The sensitivity of *helq* mutants to the topoisomerase I poison topotecan is consistent with a role for HELQ in mitotic double-strand break repair. Interestingly, flies lacking either HELQ or BLM have similarly severe SDSA defects in the *P{w^a^}* gap repair assay, but the further decrease in repair synthesis observed in the double mutant suggests that they either operate independently in SDSA or are each limiting for the same function.

**Figure 6:**
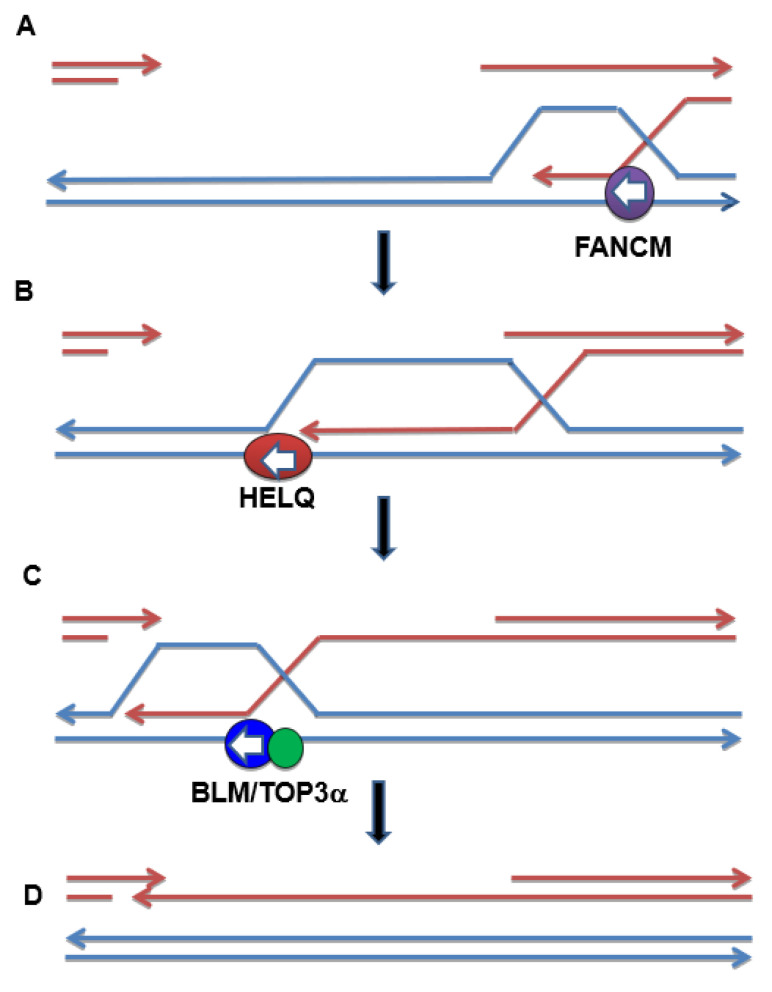
Model for the roles of the HELQ, BLM, and FANCM helicases during SDSA repair. (**A**) FANCM unwinds unproductive D-loops at the beginning of SDSA prior to the transition to processive repair synthesis. In its absence, the D-loop is processed in a way that does not predispose it to flanking deletions (not shown). (**B**) HELQ promotes enlargement of the D-loop and repairs synthesis. (**C**) As SDSA progresses, BLM-TOP3α is needed to dissociate the D-loop. In the absence of BLM, structure-specific endonucleases cleave DNA flanking the D-loop, leading to deletions (not shown). (**D**) Following D-loop dissociation, complementary ssDNA anneals and SDSA is terminated through gap filling and ligation (not shown). Arrows indicate 3′ ends of DNA. White arrowheads indicate the direction of helicase unwinding. Although only strand invasion from the right side of the gap is shown for simplicity, gap repair likely proceeds from both directions.

How might these three DNA helicases, each with 3′ to 5′ polarity, function to promote SDSA during gap repair? Of the three, only BLM is required to prevent deletions that occur when SDSA aborts and repair is presumably completed through an end-joining process [25]. Strikingly, the loss of either FANCM or HELQ partially suppresses the deletion-prone phenotype of the *blm* mutants. A possible model to explain these results could be that both FANCM and HELQ promote the early stages of SDSA. FANCM could be required to reverse unstable or unproductive D-loop structures [18] (Figure 6). In the absence of FANCM, these dead-end D-loops may need to be processed by nucleases in a way that does not create deletions flanking the original double-strand break. The synthetic lethality observed in *fancm mus81 gen1* and *fancm slx4 gen1* mutants lacking two structure-specific nucleases is consistent with this model [27]. When FANCM is present, productive D-loops are formed, and SDSA continues, with large and/or migrating D-loops that require BLM-TOP3α for their unwinding. In the absence of BLM, nucleases may act iteratively to process the extended D-loop, resulting in large flanking deletions [25]. This model can explain how the loss of FANCM rescues the deletion-prone phenotype of *blm* mutants, since SDSA intermediates that require BLM for their unwinding will not form.

What might HELQ be doing to promote SDSA? One possibility is that it could act to enlarge the D-loop, or it may be required for the transition to a mobile D-loop as repair synthesis proceeds (Figure 6). Indeed, the unwinding activity of mammalian HELQ is stimulated by the presence of RAD51, which is present at the advancing end of the D-loop [43]. In the absence of HELQ, perhaps only small or non-mobile D-loops are formed, resulting in limited repair synthesis. These D-loops would not require BLM for their dissociation, which is consistent with the suppression of deletions seen in *helq blm* double mutants.

Alternatively, HELQ might be crucial for the strand annealing stage of SDSA after the D-loop is unwound. Two recent studies identified a role for *C. elegans* and mammalian HELQ in annealing complementary ssDNA strands during microhomology-mediated end-joining and homologous recombination [43,44]. Drosophila HELQ has also been implicated in single-strand annealing repair [45]. While a putative function for HelQ in strand annealing during SDSA can potentially explain the decrease observed in the SDSA + LTR repair events in our *P{w^a^}* assay, it is more difficult to reconcile this model with the suppression of deletions observed in *helq blm* mutants. Therefore, we favor a direct role in D-loop extension for Drosophila HELQ.

Our molecular analysis of the *P{w^a^}* gap repair products suggests that synthesis normally proceeds from both ends of the gap, consistent with a mechanism where each broken end invades into the sister chromatid and repair proceeds via two-ended SDSA. However, it is also possible that repair proceeds through a break-induced replication (BIR) mechanism. Indeed, we have previously shown that the PIF1 DNA helicase, which is required for BIR in budding yeast [46,47] and promotes BIR in Drosophila [48], is needed for the efficient repair of a *P{w^a^}*-induced gap in the absence of the POL32 protein [49]. If a BIR-like mechanism is involved in gap repair in this system, then the helicases tested in the current study may also play a role in this type of repair.

In summary, we have shown that the BLM, FANCM, and HELQ DNA helicases are all required for efficient SDSA repair, with each likely playing a unique role in the process. The exact mechanisms by which each helicase promotes repair synthesis in the context of homologous recombination, and how these mechanisms interface with their other known functions in DNA repair and replication should be the focus of future studies.

## Figures and Tables

**Table 1:** Loss of HELQ partially suppresses the number of lethal flanking deletions that occur in the absence of BLM. The *P{w^a^}* construct is inserted in an intron of *scalloped* (*sd*), an *X* chromosome gene required for viability. F_1_ females inheriting an aborted SDSA repair event with a flanking deletion sometimes have malformed wings (left column), while males inheriting large deletions into the *sd* coding sequence do not survive (right column). Parenthetical numbers indicate the total number of events analyzed: left, total number of yellow-eyed F_1_ females counted; right, total number of independent yellow-eyed F_1_ females from which F_2_ males were counted. a: significantly increased from WT (*P* < 0.0001); b: significantly different from both single mutants (*P* < 0.0001); Fisher’s exact test.

Genotype	% Scalloped-Winged Females (F_1_)	% Male-Lethal Deletions (F_2_)
Wild type	0.0% (84)	0.0% (44)
*helq* ^288*A*^	0.2% (1061)	2.0% (246)
*blm^N^* ^1^	17.3% (623) ^a^	55.3% (159) ^a^
*helq* ^288*A*^ *, blm^N^* ^1^	3.9% (332) ^b^	25.2% (127) ^a,b^

**Table 2:** Increased non-lethal flanking deletions in *blm* mutants but not *helq* or *helq blm* mutants. Aberrant repair events that were negative for synthesis from the left or right end of *P{w^a^}* were analyzed for small flanking deletions into *scalloped* intron sequences. a: significantly increased from *helq* mutants (*P* < 0.05); b: significantly decreased from *blm* mutants (*P* < 0.05); Fisher’s exact test.

Genotype	Left Deletion > 100 bp	Right Deletion > 100 bp	Right Deletion > 400 bp	Right Deletion > 1200 bp
*helq* ^288*A*^	14.3% (14)	0% (10)	0% (10)	0% (10)
*blm^N^* ^1^	84.8% (33) ^a^	52.4% (21) ^a^	38.1% (21) ^a^	23.8% (21)
*helq* ^288*A*^ *blm^N^* ^1^	48.8% (43) ^a,b^	23.8% (42) ^b^	21.4% (42)	4.8% (42) ^b^

**Table 3:** Loss of FANCM partially suppresses the number of lethal flanking deletions that occur in the absence of BLM. F_1_ females with adeletion affecting the *scalloped* gene (left column) or causing lethality in males inheriting the deletion (right column). Parenthetical numbers indicate the total number of events analyzed: left, total number of yellow-eyed F_1_ females counted; right, total number of independent yellow-eyed F_1_ females from which F_2_ males were counted. The wild-type data are the same as in Table 1. a: significantly different from the wild type and *fancm* mutant (*P* < 0.00001); b: significantly different from the wild type and both single mutants (*P* < 0.05); Fisher’s exact test.

Genotype	% Scalloped-Winged Females (F_1_)	% Male-Lethal Deletions (F_2_)
Wild type	0.0% (84)	0.0% (44)
*fancm*^0693^/*Df(3R)ED6058*	1.1% (91)	0.0% (46)
*blm^D^*^2^/*blm^N^*^2^	No data	46% (69) ^a^
*fancm*^0693^*blm^N^*^2^/*Df(3R)ED6058 blm^N^*^1^	0.0% (52)	20.0% (25) ^b^

## Data Availability

The original data presented in the figures and tables of this study are available upon request from the corresponding author.

## Supplementary Materials

The following are available online at https://www.mdpi.com/article/10.3390/genes13030474/s1, Table S1: *P*-values of genotypic comparisons for SDSA + LTR annealing repair events; Table S2: *P*-values of genotypic comparisons for aborted SDSA repair events; Table S3: *P*-values of genotypic comparisons for repair synthesis tract lengths.
