# Supplementary material for: Division of Labor by the HELQ, BLM, and FANCM Helicases during Homologous Recombination Repair in Drosophila melanogaster
Source: Genes (Basel). 2022 Mar 8;13(3):474. doi: 10.3390/genes13030474 (PMC8951532; doi:10.3390/genes13030474)
Supplement: Supplementary file 1 [file genes-13-00474-s001.zip › genes-1588730-supplementary.pdf]

Supplemental Table S1:
p-values of genotypic comparisons for SDSA + LTR annealing repair events

Figure 3A

Genotype	*wild type*	*helq*	*blm*	*helq blm*	
wild type	X	< 0.0001	< 0.0001	< 0.0001	
helq	X	X	> 0.99	0.55	
blm	X	X	X	0.04	
helq blm	X	X	X	X	

Figure 4A

Genotype	*wild type*	*fancm*	*blm*	*blm fancm*	
wild type	X	< 0.0001	< 0.0001	< 0.0001	
fancm	X	X	0.005	0.10	
blm	X	X	X	0.96	
blm fancm	X	X	X	X	

Figure 5A

Genotype	*wild type*	*helq*	*fancm*	*helq fancm*	
wild type	X	<0.0001	<0.0001	<0.0001	
helq	X	X	0.20	0.18	
fancm	X	X	X	0.99	
helq fancm	X	X	X	X	

p-values were calculated with the Kruskal-Wallis test with Dunn's multiple comparisons, using GraphPad Prism 9 software. Comparisons with p<0.05 are highlighted in yellow.

Supplemental Table S2:
p-values of genotypic comparisons for aborted SDSA repair events

Figure 3B

Genotype	*wild type*	*helq*	*blm*	*helq blm*	
wild type	X	< 0.0001	< 0.0001	< 0.0001	
helq	X	X	0.006	0.19	
blm	X	X	X	> 0.99	
helq blm	X	X	X	X	

Figure 4B

Genotype	*wild type*	*fancm*	*blm*	*blm fancm*	
wild type	X	0.25	< 0.0001	0.0001	
fancm	X	X	< 0.001	0.0083	
blm	X	X	X	0.89	
blm fancm	X	X	X	X	

Figure 5B

Genotype	*wild type*	*helq*	*fancm*	*helq fancm*	
wild type	X	< 0.0001	0.63	0.005	
helq	X	X	< 0.0001	0.48	
fancm	X	X	X	0.03	
helq fancm	X	X	X	X	

p-values were calculated with the Kruskal-Wallis test with Dunn’s multiple comparisons, using GraphPad Prism 9 software. Comparisons with $p<0.05$ are highlighted in yellow.

Supplemental Table S3:
p-values of genotypic comparisons for repair synthesis tract lengths

Figure 3C

Genotypic comparison	Minimum number of base pairs synthesized				
	5	250	1000	2400	4600
wild type / helq	0.29	0.14	0.0002	0.0042	0.0004
wild type / blm	0.0012	0.0023	< 0.0001	< 0.0001	0.033
helq / blm	0.044	0.14	0.23	0.0015	0.210
helq / helq,blm	< 0.0001	< 0.0001	< 0.0001	< 0.0001	0.68
blm / helq,blm	0.0004	< 0.0001	0.0001	0.026	0.097

Figure 4C

Genotypic comparison	Minimum number of base pairs synthesized				
	5	250	1000	2400	4600
wild type / fancm	0.11	0.12	0.038	0.15	0.13
wild type / blm	0.064	N.D.	< 0.0001	< 0.0001	0.011
fancm / blm	1.0	N.D.	0.098	0.0026	0.43
fancm / blm,fancm	1.0	0.13	0.0069	0.0068	1.0
blm / blm,fancm	1.0	N.D.	0.21	1.0	1.0

Figure 5C

Genotypic comparison	Minimum number of base pairs synthesized				
	5	250	1000	2400	4600
wild type / fancm	0.11	0.12	0.038	0.15	0.13
wild type / helq	0.29	0.14	0.0002	0.0042	0.0004
fancm / helq	0.58	0.81	0.40	0.41	0.23
helq / helq,fancm	0.80	< 0.0001	< 0.0001	< 0.0001	0.26
fancm / helq,fancm	0.79	0.0019	< 0.0001	< 0.0001	0.025

Two-tailed p-values were calculated with Fisher's exact test, using GraphPad Prism 9 software. N.D. = no data. Comparisons with $p<0.05$ are highlighted in yellow.
